# The heterotopic heart transplantation in mice as a small animal model to study mechanical unloading – Establishment of the procedure, perioperative management and postoperative scoring

**DOI:** 10.1371/journal.pone.0214513

**Published:** 2019-04-12

**Authors:** Sumi Westhofen, Marisa Jelinek, Leonie Dreher, Daniel Biermann, Jack Martin, Helga Vitzhum, Hermann Reichenspurner, Heimo Ehmke, Alexander Peter Schwoerer

**Affiliations:** 1 Department of Cardiovascular Surgery, University Heart Center, Hamburg, Germany; 2 DZHK (German Centre for Cardiovascular Research), partner site Hamburg/Kiel/Lübeck, Hamburg, Germany; 3 Department of Cellular and Integrative Physiology, University Medical Center Hamburg Eppendorf, Hamburg, Germany; 4 Department of Surgery, Addenbrookes Hospital, University of Cambridge, Cambridge, United Kingdom; Imperial College Healthcare NHS Trust, UNITED KINGDOM

## Abstract

**Background:**

Unloading of failing hearts by left ventricular assist devices induces an extensive cardiac remodeling which may lead to a reversal of the initial phenotype–or to its deterioration. The mechanisms underlying these processes are unclear.

**Hypothesis:**

Heterotopic heart transplantion (hHTX) is an accepted model for the study of mechanical unloading in rodents. The wide variety of genetically modified strains in mice provides an unique opportunity to examine remodeling pathways. However, the procedure is technically demanding and has not been extensively used in this area. To support investigators adopting this method, we present our experience establishing the abdominal hHTX in mice and describe refinements to the technique.

**Methods:**

In this model, the transplanted heart is vascularised but implanted in series, and therefore does not contribute to systemic circulation and results in a complete mechanical unloading of the donor heart. Training followed a systematic program using a combination of literature, video tutorials, cadaveric training, direct observation and training in live animals.

**Results:**

Successful transplantation was defined as a recipient surviving > 24 hours with a palpable, beating apex in the transplanted heart and was achieved after 20 transplants in live animals. A success rate of 90% was reached after 60 transplants. Operative time was shown to decrease in correlation with increasing number of procedures from 200 minutes to 45 minutes after 60 operations. Cold/warm ischemia time improved from 45/100 to 10/20 minutes. Key factors for success and trouble shootings were identified.

**Conclusion:**

Abdominal hHTX in the mouse may enable future examination of specific pathways in unloading induced myocardial remodeling. Establishment of the technique, however, is challenging. Structured training programs utilising a variety of training methods can help to expedite the process. Postoperative management, including daily scoring increases animal wellbeing and helps to predict survival.

## Introduction

Implantation of left ventricular assist devices (LVADs) is regularly used in patients with end-stage heart failure, either as a bridge to transplantation, as a bridge to recovery, or as a destination therapy.[1] LVADs increase cardiac output and strongly reduce the cardiac workload. In some patients, this mechanical unloading may induce a reverse remodeling with beneficial consequences on ventricular geometry, myocardial structure, contractility and pump function.[2–9] On the other hand, mechanical support has also been reported to provoke myocardial atrophy and fibrosis, and to impair cardiac electrophysiology and calcium handling.[10–16] The mechanisms underlying the beneficial and the detrimental effects of LVADs on cardiac physiology are poorly understood.

The fully unloaded heterotopic heart transplantation (hHTX) in rodents is an internationally accepted animal model which is mainly used to investigate transplantation biology.[17–30] In this experimental model, the heart of a donor animal is heterotopically transplanted in a recipient animal. Due to the configuration of the anastomoses, the graft beats with markedly reduced left ventricular filling while coronary perfusion is preserved. Thus, the hHTX is a suitable model to study unloading associated remodeling. The first heterotopic abdominal heart transplantation was published using rats by Abbott et al. in 1964.[31] Following its modification by Ono et al. a couple of years later, it has been widely adopted as a rodent model.[17, 18, 32–40] Notably, most studies using the hHTX addressing unloading induced cardiac remodeling have been performed in rats. Compared to mice, genetic modifications in rats are limited which reduces the possibilities of testing mechanistic hypotheses. The hHTX in mice, however, is technically highly demanding with a low error tolerance. Establishing the technique is, therefore, time-consuming and costly which may prevent laboratories from adopting the hHTX.

To support investigators learning the hHTX in mice, we here present our experiences establishing this method following several years performing the hHTX in rats. We provide details of the operation procedure and of the perioperative management. Furthermore, we present several aspects of trouble shooting and a scoring protocol for increased postoperative animal care.

## Materials and methods

**Fig 1:**
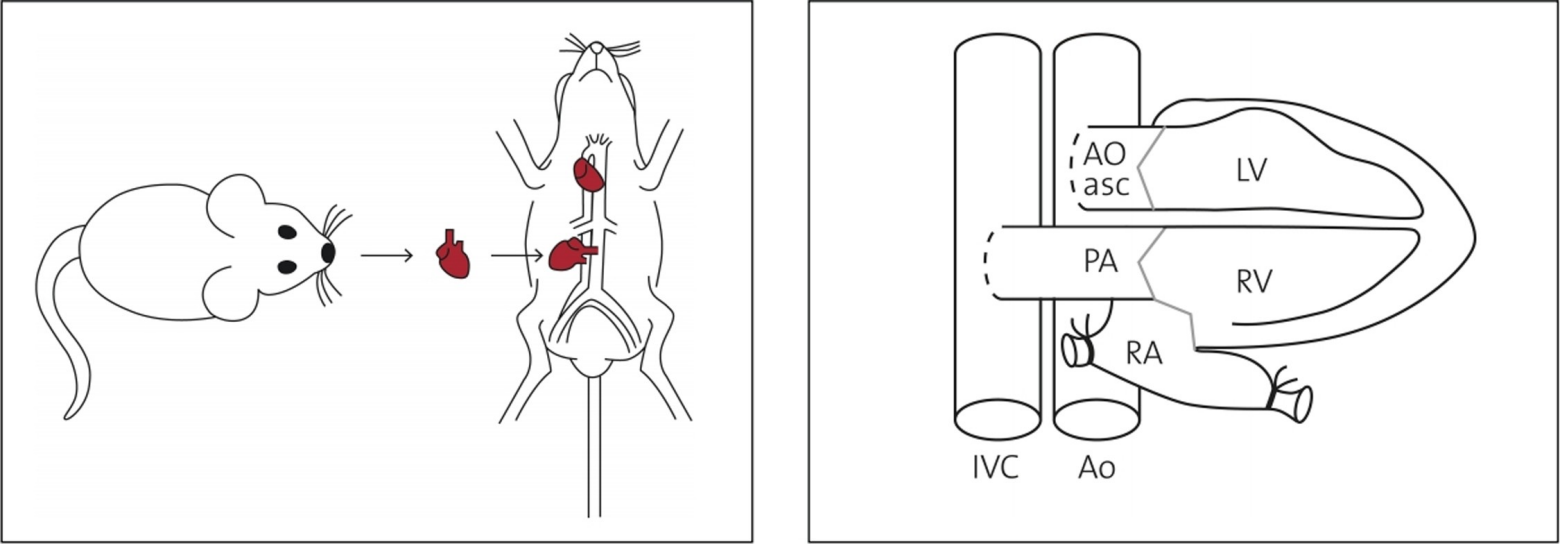
**Schematic illustration of the heterotopic abdominal heart transplantation (a) and of the anastomoses (b).** The donor ascending aorta (Ao asc) was anastomosed to the recipient infrarenal aorta (Ao), and the donor pulmonary artery (PA) was anastomosed to the recipient inferior vena cava (IVC). Due to the absence of atrial filling, and in the presence of competent aortic and pulmonary valves, the left and the right ventricle (LV, RV) are mechanically unloaded.

The transplantation technique has previously been reported and leads to complete mechanical unloading of the donor heart (Fig 1A).[17, 18, 41] Briefly, the heart of a donor animal is explanted, preserved in cooled saline solution, and transplanted in the abdomen of a recipient animal. The donor ascending aorta is anastomosed to the recipient infrarenal aorta, and the donor pulmonary artery is anastomosed to the recipient inferior vena cava (IVC, Fig 1B).

To establish the hHTX in mice, an educational program was devised with discrete learning objectives utilizing a range of teaching methods [15–18, 28, 42]. Using a targeted approach, the transplant establishment was broken down into a series of steps with the aim of improving efficiency in the acquisition of the technical skills required to perform the operation.

Step 1: Current literature and video tutorials were reviewed, and key factors from these technical descriptions that influenced outcome were identified.

Step 2: The hHTX was observed and performed in rats to develop microsurgical skills. This step was performed in our own institution that has extensive experience in rat hHTX.

Step 3: Initial technical skills were developed in mice on cadaveric specimens with the donor and recipient steps of the operation performed using a single animal. When the donor procedure was completed in less than 20 minutes and the recipient procedure performed in less than 120 minutes the surgeon would progress to step 5.

Step 4: A mentorship program was undertaken with two other institutions that had successfully established the procedure in mice.

Step 5: The procedure was performed in live animals when the operation was performed in cadaveric specimens in less than 140 minutes. For the live procedure two animals were used–a donor and a recipient animal.

### Animals

Male and female FVB mice with a mean age of 7–12 weeks weighing 16-35g were acquired from our animal facility. Mice were maintained in specific pathogen-free animal facilities with ad libitum access to food and water at the Institute of Cellular and Integrative Physiology, University Medical Center Hamburg. The animal study was reviewed and approved by the local authority for animal protection (Behörde für Gesundheit und Verbraucherschutz Hamburg, Approval No. A8a/785 and G13/098). All experiments were performed in accordance with the German legislation on the protection of animals.

### Preparations of donor and recipient animals

Thirty minutes before the induction of anesthesia, 0.1 mg/kg of buprenorphine and 5.0 mg/kg of carprofen were injected intraperitoneally (i.p.). The inhalative anesthesia (isoflurane, 3% during the induction and 1.5% during the maintenance of the anesthesia) was delivered via a nose cone from an anesthesia device system. Animals were placed in a supine position and the body temperature was maintained at 37°C with the use of a heating pad (TR-200, Fine Science Tools, Heidelberg, Germany). The operative procedure was performed using a dissection microscope (WILD Heerbrugg 355110, Leica) with 6–25 -fold optical magnification.

### Donor operation

**Fig 2:**
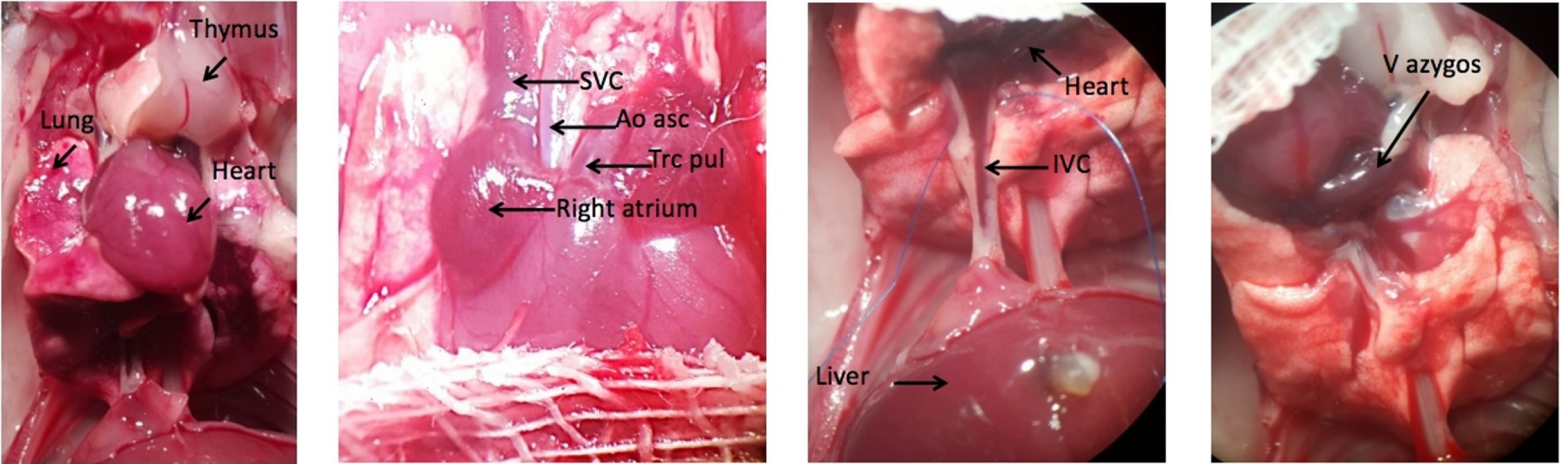
**a-d: Photographic illustrations of the donor operation**. Fig 2A shows the donor heart in-situ with the thymus turned upwards. Fig 2B is a close-up of the heart and its superior vessels: the superior vena cava (SVC), ascending aorta (Ao asc). The SVC is being ligated, the Ao asc is transected just below the brachiocephalic artery, and the pulmonary artery (Trc pul) is transected just below its bifurcation. Fig 2C shows the inferior vena cava (IVC) before its ligation. Fig 2D shows the azygos vein, which is also being ligated, together with the pulmonary veins.

After a midline abdominal incision was made, as much blood as possible (0.5–0.8 ml) was aspirated with a 1-ml syringe and a 30-gauge needle from the IVC to reduce cardiac preload. Then, 0.5 ml of ice-cold heparin solution (100 U ml^-1^) was injected into the IVC using a 30-gauge needle. Following a short delay, the abdominal aorta was punctured in order to prevent volume overloading of the heart. After 1 minute for the systemic heparinization, the abdominal incision was extended towards the thoracic inlet by cutting along both sides of the thoracic spine. The mobilized anterior chest wall was reflected superiorly. The thymus was resected (Fig 2A) to aid access to the aortic arch. The aortic arch was dissected and 0.5 ml of ice-cold heparin solution was slowly injected using a 30-gauge needle. Then the superior and inferior venae cavae were ligated using 8–0 silk sutures. Afterwards, the ascending aorta was transected below the brachiocephalic artery, and the main pulmonary artery was transected proximal to its bifurcation (Fig 2B and 2C). Connective tissue between the ascending aorta and the pulmonary artery was carefully dissected. Finally, the pulmonary veins and the azygos vein were ligated as a group with a single 8–0 silk suture (Fig 2D). Carefully, the graft was bluntly dissected from the remaining connective tissue. Until transplantation, the heart was preserved in ice-cold saline solution.

### Recipient operation

**Fig 3:**
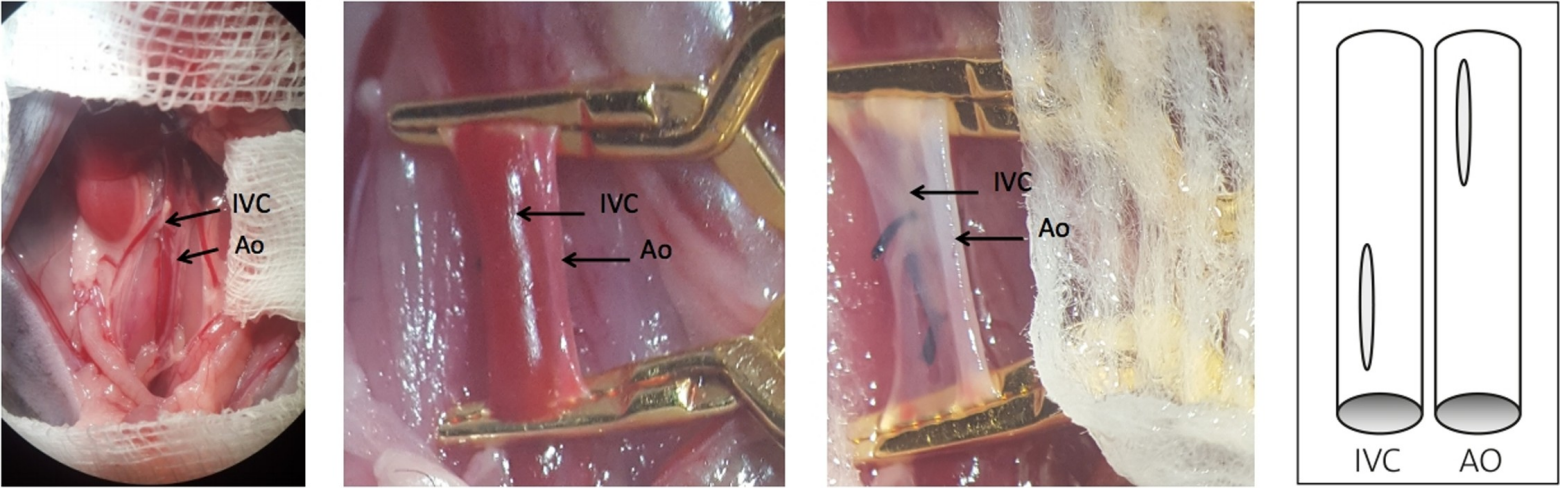
Photographic illustration of the recipient situs. Fig 3A shows the infrarenal abdominal aorta (Ao) and the inferior vena cava (IVC). In the left upper corner, the right kidney can be identified. At the lower border the reproductive organs can be found, dorsally of the reproductive organs is the iliac bifurcation. Fig 3B shows the Ao and the IVC after clamping. Fig 3C shows the Ao and the IVC after aortotomy and venotomy, and flushing of the vessels. Fig 3D shows a schematic illustration of the aortotomy (infra-renal abdominal aorta) and venotomy (subrenal IVC) after clamping the vessels.

The abdomen of the recipient animal was opened with a midline incision from the pubis to the xiphoid. Using a retractor, the abdominal cavity was exposed. The intestines were carefully moved cranially and to the sides without removing them from the abdomen. The liver and the intestines were covered with a gauze drenched in warm saline. The reproductive organs and the bladder were also carefully moved to the sides and covered with a gauze drenched in warm saline (Fig 3A). Infrarenally, the abdominal aorta and the IVC were carefully dissected, and a group of centrally positioned lumbal vessels were ligated with a 8–0 silk suture. Using two clips (Yasargil Clip, Aesculap, Inc.–a.B. Braun company, Center Valley, PA, USA), the blood flow through the abdominal aorta and the IVC was interrupted. The first clip was placed proximally of the iliac bifurcation and the second one just distally of the renal vessels (Fig 3B). Meticulous ligation of all lumbar vessels was performed between the two clips. Using a 30-gauge needle, an aortotomy was made at the proximal end of the clamped aorta, and a venotomy was performed at the distal end of the clamped IVC (Fig 3C and 3D). Both vessels were flushed with ice-cold saline solution until no residual blood was left in the clipped section. The aortotomy was extended distally and the venotomy proximally in a longitudinal orientation, using fine scissors.

### Heterotopic heart transplantation

**Fig 4:**
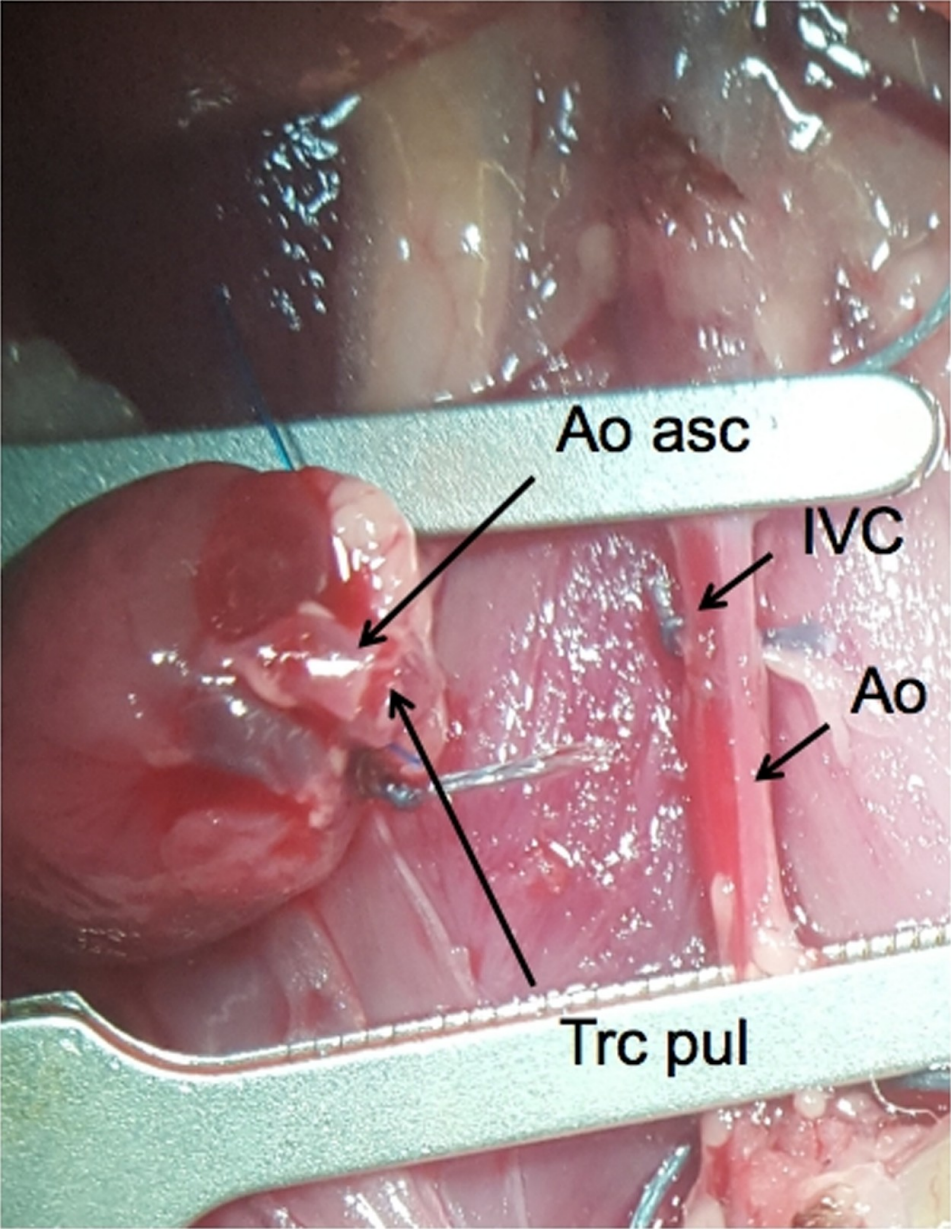
The recipient situs with clamped abdominal vessels. The abdominal aorta (Ao) and the inferior vena cava (IVC) were clamped and prepared for the anastomoses. The donor heart is already situated next to the vessels with the ascending aorta (Ao asc) and the truncus pulmonalis (Trc pul) in place.

**Fig 5:**
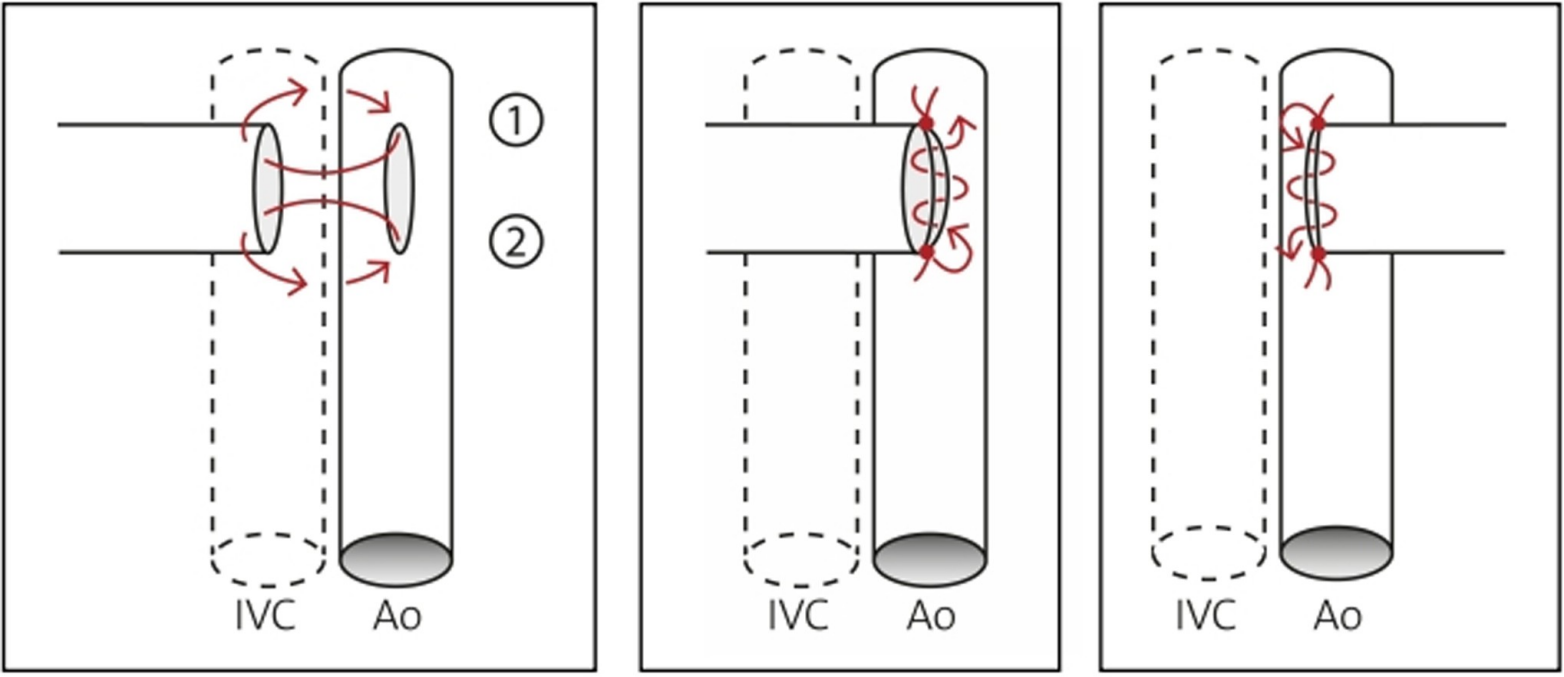
**a-c: Schematic illustration of the arterial anastomosis on the abdominal aorta (Ao), while the broken line indicates the inferior vena cava (IVC).** Fig 5A shows the proximal and distal anchor stitch. Fig 5B shows the continuously running suture on the left side from the distal anchor stitch towards the proximal anchor stitch. Fig 5C shows the right side of the arterial anastomosis after the heart was carefully flipped over to the left side of the abdomen.

**Fig 6:**
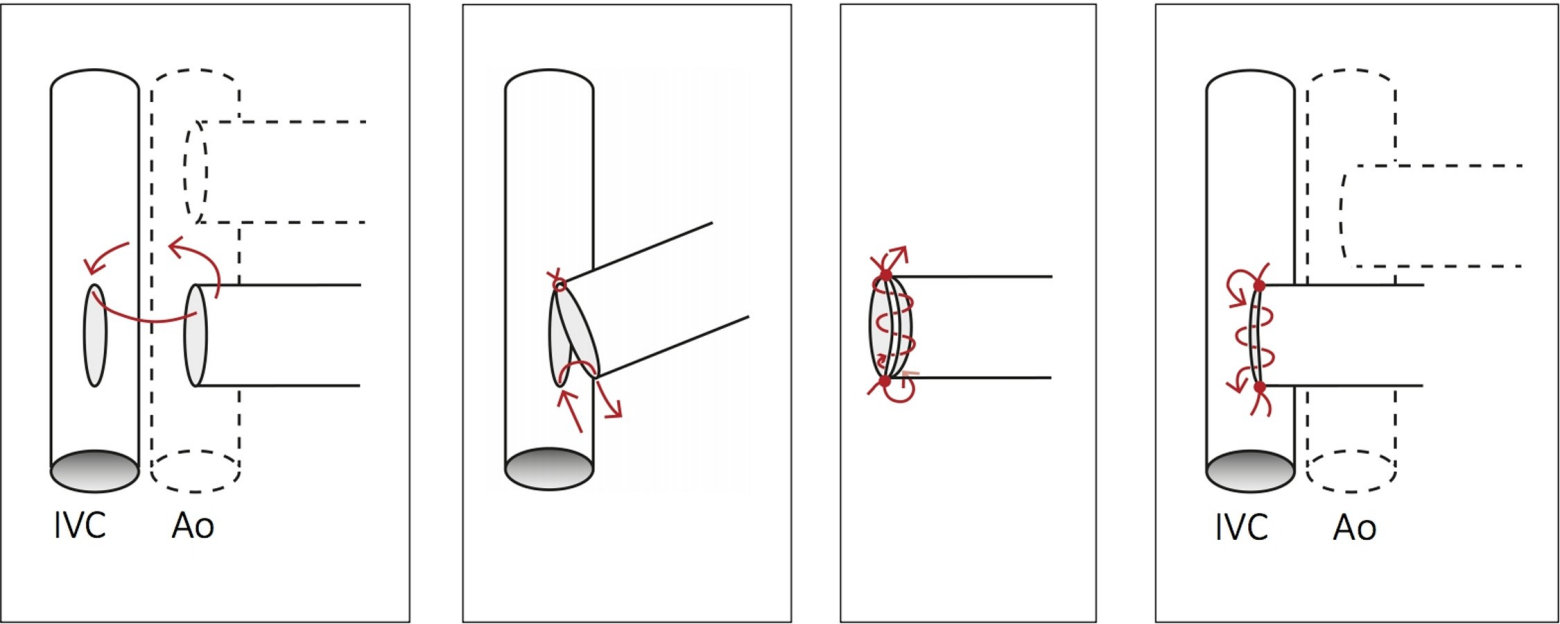
**a-d: Schematic illustration of the venous anastomosis on the inferior vena cava (IVC), while the broken line indicates the abdominal aorta (Ao).** For the venous anastomosis, also proximal and distal anchor stiches were performed (Fig 6A and 6B). Fig 6C shows how after the distal anchor stitch was performed, the suture was pulled through outwardly between the pulmonary artery and the inferior vena cava (IVC), so the continuous suture could then be started on the right side of the anastomosis, stitching from outside-to-inside and inside-to-outside (5–7 stiches). Fig 6D shows the left side of the anastomosis.

**Fig 7:**
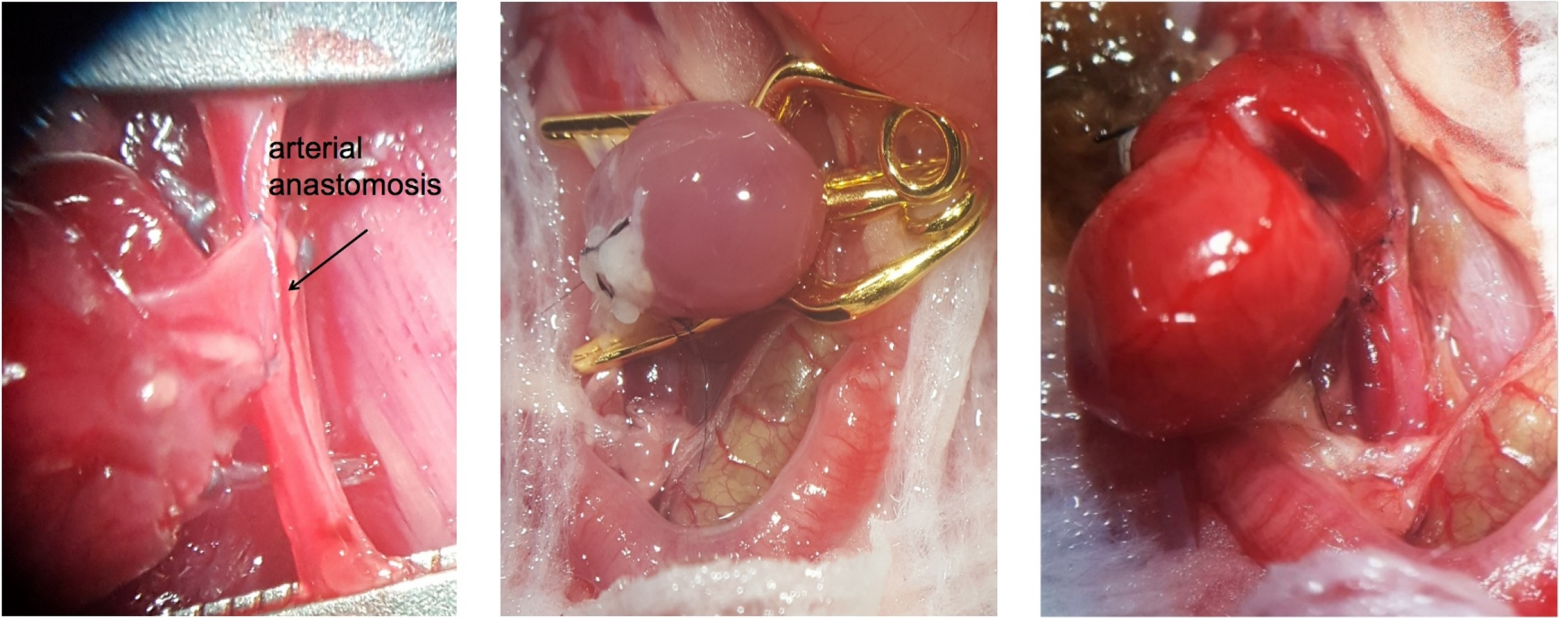
**a-c: Photographic illustration of the heterotopic transplantation**. Fig 7A shows the finished left side of the arterial anastomosis, afterwards the heart is being flipped over for the right side of the arterial anastomosis. Fig 7B and 7C show the transplanted heart after finishing all anastomoses. Fig 7B shows the transplanted heart before de-clamping. Fig 8C shows the heart which is directly perfused following removal of the clamps.

The donor heart was taken out of the cold preservation solution and placed on the right side of the abdominal IVC. The ascending aorta and the pulmonary artery of the graft were orientated perpendicular to the IVC and abdominal aorta of the recipient animal. The ascending aorta was positioned ventral to the pulmonary artery (Fig 4). The donor heart was then covered with gauze drenched in ice-cold saline. The heart was regularly cooled (every 3–5 mins) by topical application of ice-cold saline solution whilst the anastomosis was performed. First, the arterial anastomosis was performed, starting with an anchor stitch placed proximally, then distally, with a 10–0 suture (Fig 5A). After knotting the distal anchor stitch, a continuously running suture of 4–6 stitches on the left side towards the proximal anchor stitch was performed (counter-clockwise direction, from outside-to-inside and inside-to-outside), and the suture was tied to the proximal anchor stitch (Figs 5B and 7A). The heart was then carefully flipped over to the left side of the abdomen, and the right side of the arterial anastomosis was finished with 4–6 stitches, and the suture was tied to the distal anchor knot (Fig 5C). For the venous anastomosis, again two anchor stitches were positioned, first proximally, then distally (Fig 6A and 6B). After the distal anchor stitch was performed, the suture was pulled through to the outside of the vessel, between the pulmonary artery and the IVC, so the continuous suture could then be started on the right side of the anastomosis, stitching from outside-to-inside and inside-to-outside (Fig 6C). Using this approach, the heart did not have to be flipped over a second time, improving economy of movement and reducing the strain on the vessels. After tying the suture to the proximal anchor knot, the left side of the anastomosis was finished (Fig 6C and 6D). For the venous anastomosis, 5–7 stitches were made on each side. The suture was not tightened firmly and a loop-suture was left in the middle of the first anastomotic side, to prevent congestion.

After finishing both anastomoses, warm saline was poured over the heart, and small stripes of a hemostatic agent (Tabotamp, Ethicon, Inc., Somerville, NJ, USA) were draped around the anastomoses. The distal clip was removed first allowing slow retrograde filling of the heart (Fig 7B and 7C) before the proximal clip was released. If necessary, additional hemostasis using cotton swabs with light pressure was performed. Typically, the heart would beat spontaneously after a short period of fibrillation. The initial bradycardic heart rate recovered after about 1–2 hours. This process was supported by warming the abdomen with topical application of warmed saline. The intestines were repositioned avoiding torsion, and the laparotomy closed (subcutaneous suture and skin suture) with a continuous 5–0 nylon suture.

**Fig 8:**
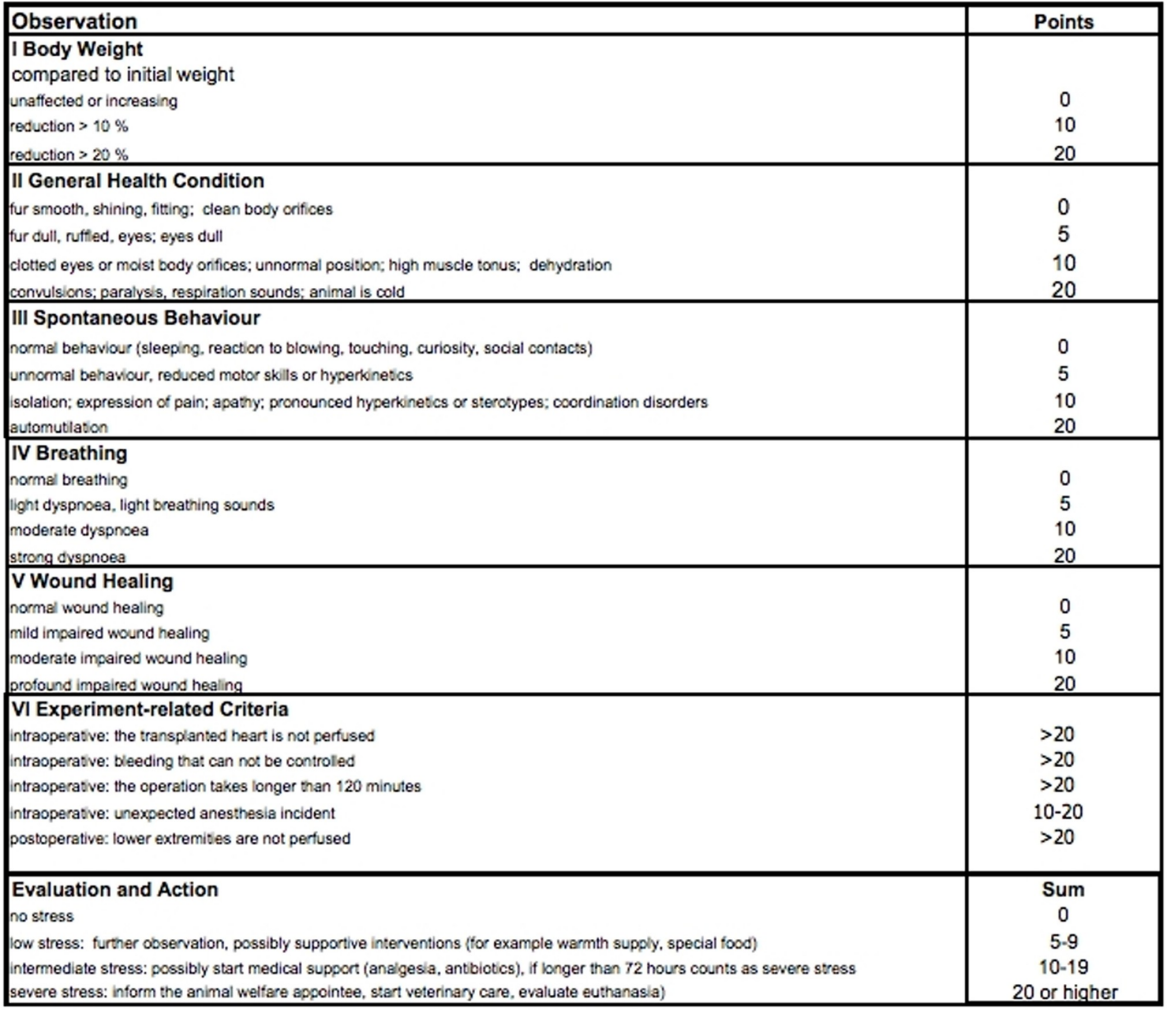
Scoring sheet used for daily postoperative evaluation of the recipient animal. Five categories were used to score the animals postoperatively. For each category points were assigned, that were summed up in a total score to evaluate if further action was required.

### Postoperative care and scoring

After the operation, the recipient mouse was placed in a warming cage with access to food and water ad libitum. Postoperative care and observation of the operated animals were performed after 1, 3, 6, 8 and 12 hours. Afterwards, animals were scored on a daily basis, including five parameters: body weight, general health condition, spontaneous behavior, breathing and wound healing (Fig 8). For each criterium 0 to 20 points could be assigned and the total score was calculated each day to decide on whether further action was required. A score of 0 implicated no stress and no further action was necessary. At a score of 5–9 (low stress) further observation and possibly supportive interventions (e.g. warmth supply) were indicated. At a score of 10–19 (intermediate stress), a start of medical support (analgesia, antibiotics) had to be discussed. If this score persisted longer than 72 hours, it was defined as a severe stress level. At a total score of ≥ 20 (severe stress), the animal welfare appointee had to be informed and intensified veterinary care had to be started. Furthermore, euthanasia had to be evaluated. Pain medication was administered daily (every 8 hours during the first 3 postoperative days) for the first 8 postoperative days using buprenorphine (0.1 mg/kg KG s.c.) and metamizole (300 mg/kg KG p.o.) Furthermore, the transplanted graft was assessed daily via palpation (presence or absence of regular contractions).

### Statistics

Continuous variables are presented as mean ± standard deviation, or median and quartiles, and categorical variables are presented as percentage. Comparisons of continuous variables that were normally distributed were performed with the Students t-test, comparison of continuous variables that were not normally distributed were performed with the Mann-Whitney-U-Test. Statistical software used was Prism 7.0c

## Results

### Learning curve

**Fig 9:**
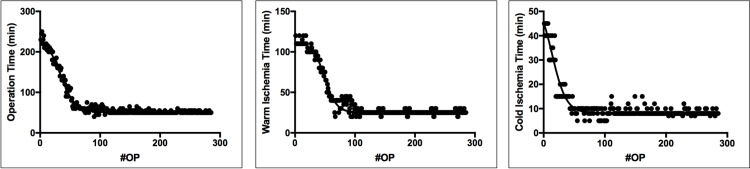
**a-c: Graphical illustration of the learning curve: Operation times**. Total operation time (a), warm ischemia time (b), and cold ischemia time (c) with time in minutes on the y-axis and number of operations on the x-axis (#OP).

**Fig 10:**
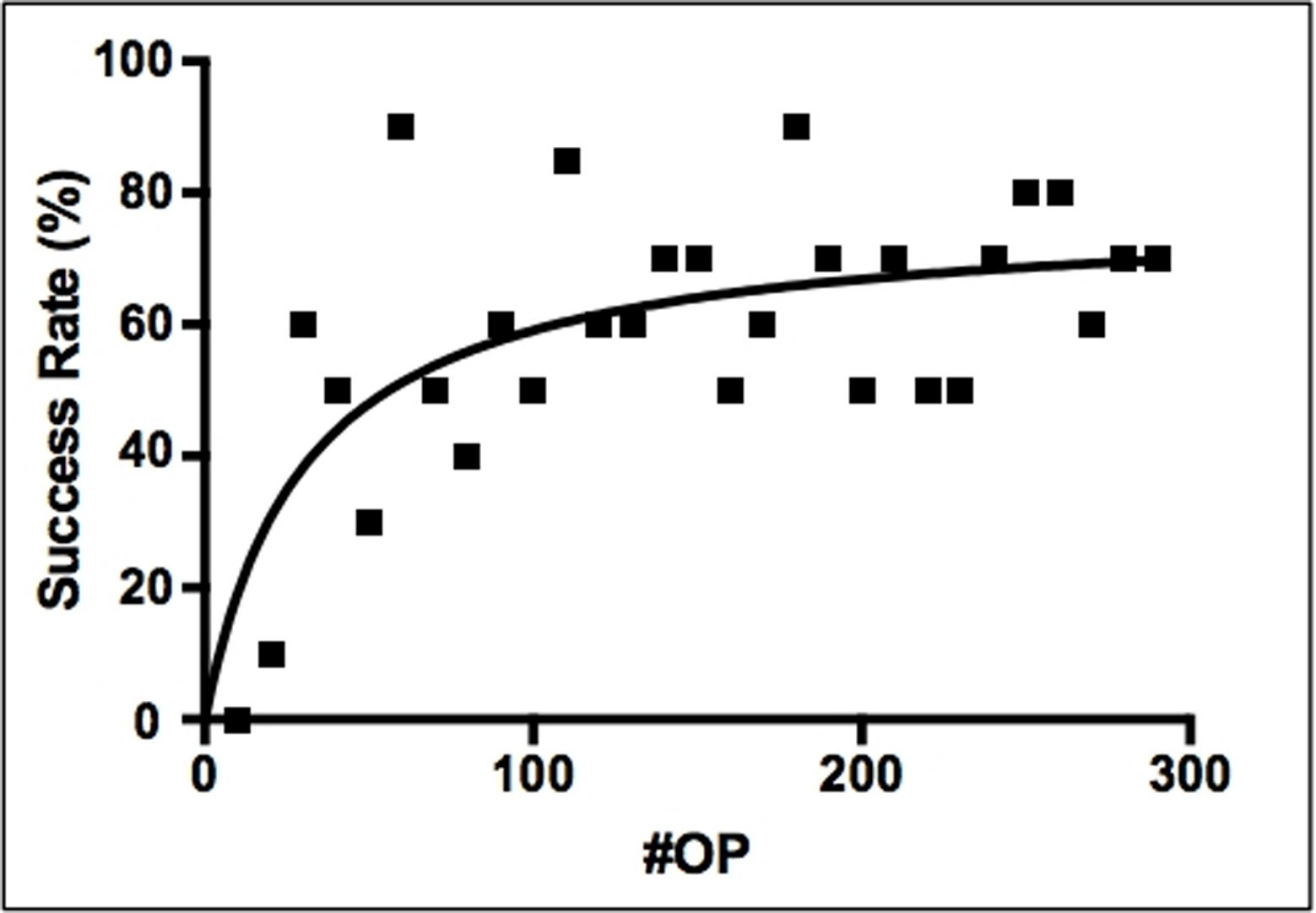
Graphical illustration of the learning curve: Success rate. Success was defined by a well-beating donor-heart after transplantation and survival of the recipient ≥48 hours.

In a first step, theoretical knowledge was acquired, using literature research and video education, and first practical training was then performed in cadaveric procedures. Before practical training using live specimens was commenced, in a mentorship program with other institutions that had successfully established the method, technical advice and skills were acquired. Our experience on hHTX in alive mice is based on a total of 286 procedures, all performed by one surgeon, between December 2015 and July 2017. Twenty cadaveric procedures were performed to reach technical confidence. The first successful transplantation with a well-beating donor-heart and survival of the recipient ≥48 hours was achieved after 20 live procedures. Total operation time decreased in correlation with increasing number of procedures from 250 minutes to 45 minutes after 60 live transplantations (Fig 9A). Cold ischemia time improved in the early training phase from 45 to 8 minutes, and warm ischemia time from 120 to 25 minutes (Fig 9B and 9C). Operation time and ischemia times stabilized after ~60 procedures. A first success rate of 90% was reached after 60 live transplantations (Fig 10). Notably, even though the operation time which can be interpreted as a gross marker for the complication rates was stable, survival rates of the following operations had a strong variation (Fig 10). In the early training phase (<60 procedures) different mice characteristics (e.g. age, weight) contributed to fluctuating success rates. Heterogeneity in individual responses to unloading was also observed, especially in the early learning phase, leading to varying degrees of heart weight reduction post-transplantation (data not shown). This suggests secondary processes, i.e. valvular leakage, in some transplants and underlines the complexity of this procedure. Procedural semiquantitative scores for intraoperative bleeding complications, time until rebeating, contractility, rhythm within the first minutes after reperfusion, congestion and anastomosis problems, and any other technical complication were collected. Key factors for success and trouble shootings establishing this operation were identified.

### Postoperative scoring

**Fig 11:**
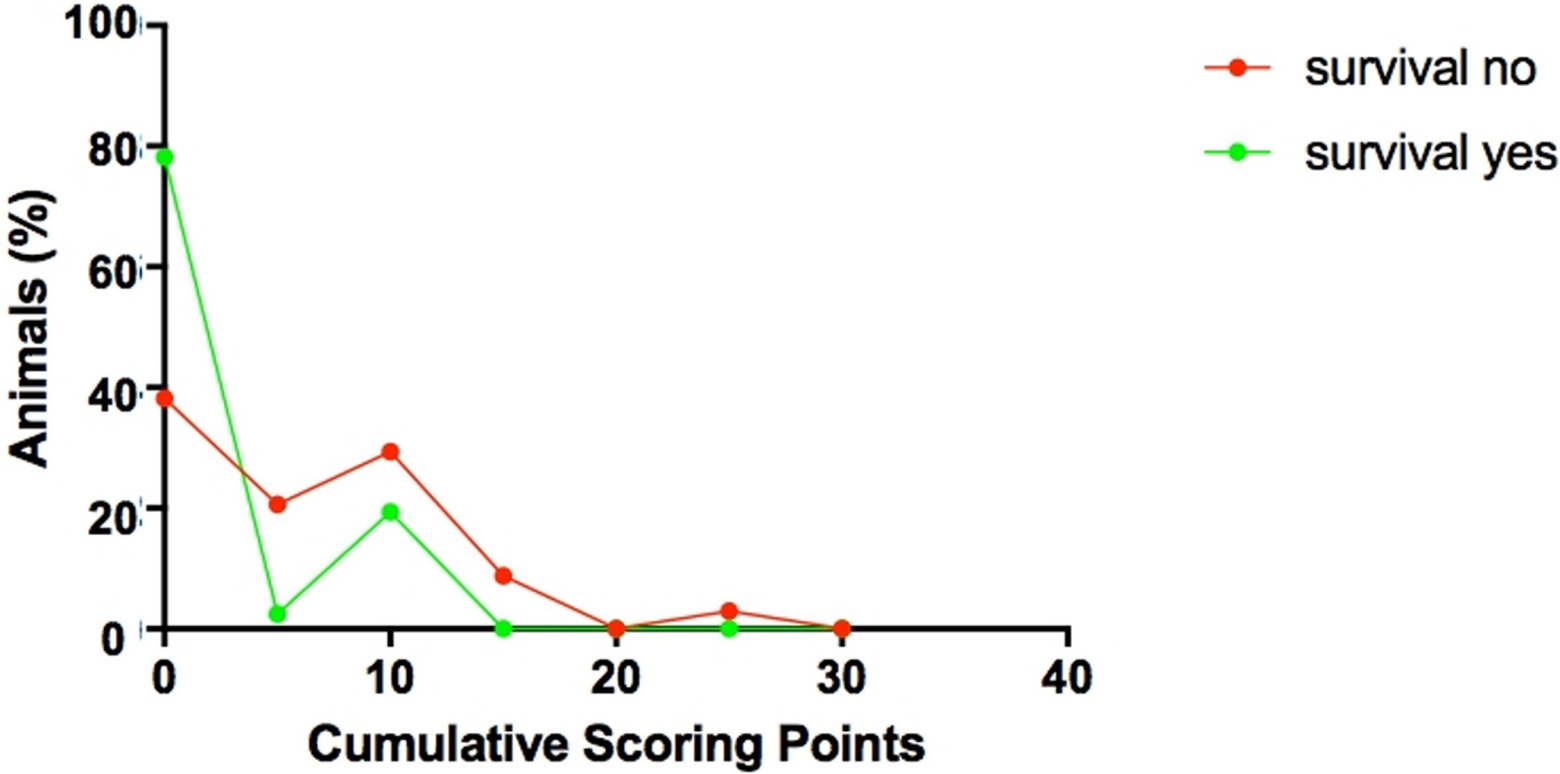
Graphical illustration showing the correlation between postoperative total scoring points and survival. A comparison of the green curve (survival yes) and the red curve (survival no) shows that more animals (y-axis) with a lower score (x-axis) survived.

**Fig 12:**
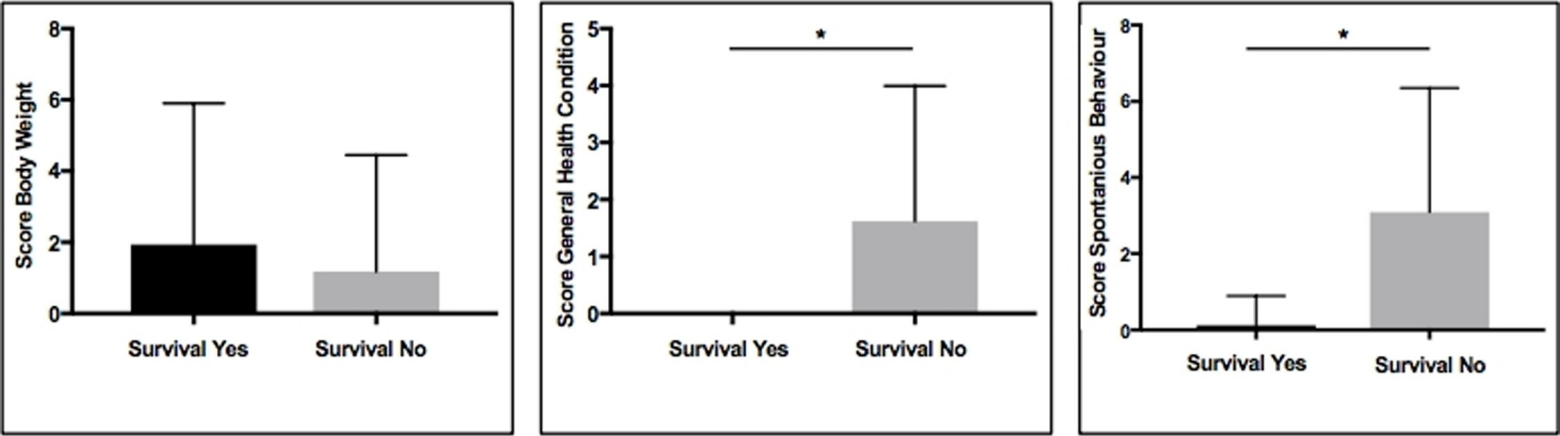
**a-c: Graphical illustration showing the correlation between postoperative scoring parameters and survival.** Displayed are in detail body weight (a), general health condition (b) and spontaneous behavior (c), with the mean score on the y-axis and survival on the x-axis.

121 (42.3%) of the 286 operated recipient mice did not survive the planed observation period of ≥ 48 hours. Of these, 87 (72.0%) died during the first 12–24 hours after the operation, and could, therefore, not be included in the postoperative scoring routine which started 24 hours following operation. When animals showed signs of stress during postoperative observation and scoring was positive (≥20 points), supportive interventions like application of warmth, fluid injection (warm saline solution, i.p., 0.5ml), administration of pain medication or antibiotics (baytril) were performed. In the majority of cases, supportive interventions did not lead to an improved survival and therefore success. As expected, scoring analysis revealed that a lower total postoperative score correlated with better survival (P<0.001, Fig 11). Notably, 80% of the surviving animals had a score of 0. In a subgroup analysis of the five scoring parameters, only the scores relating to the spontaneous behavior and to the general health condition showed a significant negative correlation with survival (P<0.001; Fig 12). Weight loss did not predict survival, as e.g. 20% of the surviving animals had a score of 10 due to weight loss. We saw no case of breathing disorder, impaired wound healing or paraplegia.

### Gender analysis

**Fig 13:**
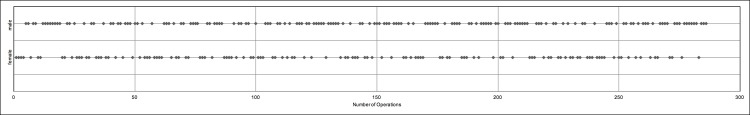
Graphical illustration of gender distribution over time. The number of operations over time are displayed on the x-axis, with gender (male/female) on the y-axis. Each datapoint represents one operation.

Operations of male and female mice were performed alternatingly over time (Fig 13). 162 (56.6%) of the 286 operations were performed in male mice vs. 124 (43.4%) in female mice. 81 (50.0%) of the male-mouse-transplantations were successful vs. 81 (65.3%) of the female-mouse-transplantations (P = 0.002). With regards to postoperative scoring, there was no statistically significant difference between the genders (P = 0.765). Male animals displayed a more stable heart weight reduction following unloading (data not shown).

### Procedural scores

Overall, intraoperative bleeding problems at the anastomotic side occurred in 43.3% during the first 60 live transplantations, mostly due to technical inaccuracy since the exact placement and spacing of stitches is essential for operative success. It was possible to reduce intraoperative bleeding problems to 24.7% during the following 226 operations (P<0.01). Retrograde bleeding due to insufficient ligation of lumbar vessels was observed in 26.6% during the first 60 live transplantations, and in 20.8% during the following 226 operations. This was usually noticed directly after incision of the recipient vessels, and therefore could be corrected before the blood loss was too severe. Early congestion of the graft was observed in 23.3% during the first 60 live transplantations, and in 15.5% during the following 226 operations. Late thrombosis of the graft in 11.7% during the first 60 live transplantations, and in 7.5% during the following 226 operations, mostly due to a too tight venous anastomosis. When anastomoses were technically accurate, grafts started to beat immediately after reperfusion. Usually, a short period of fibrillation was observed during the reperfusion phase. Recovery of normal contractility correlated with the quality of the anastomoses and short ischemia times. Hindlimb paralysis was only observed in one very early operation trying to use an electro-cauter for interrupting the blood flow from the lumbar vessels. The use of an electro-cauter had not been problematic in rats in our experience but was discontinued in mice.

## Discussion

The hHTX in mice can be used to study cardiac remodeling in mechanically unloaded hearts and to address underlying mechanisms. Since its first description by Corry et al. in 1973, the model has been in widespread use and various modifications have been published.[34, 40] This underlines the technical complexity but also the value of this model. Different modifications of operative steps, pitfalls and troubleshooting have already been published, however the establishment of the technique remains challenging.[17, 18] To encourage working groups to use this animal model, and to reduce initial failures, we presented here our own experiences with emphasis on a structured educational outline, on technical aspects & trouble shooting and on the postoperative management.

### Educational program

Our educational program consisted of five consecutive steps: (1), starting with a review of the literature and video education, a theoretical understanding was established. (2), watching and performing the hHTX in rats allowed to get a first practical experience. However, due to the considerable differences in size, the translation to mice was limited. In our view, this step was of limited value. (3), cadaveric procedures were performed to gain technical confidence. This step is of great value, since it reduces harm in alive animals. Switch to live procedures should only be performed when the sequence of the procedure and the sutures are well established. (4), experience from other working groups who already established the technique in mice was shared during laboratory excursions of several day’s duration. Here, the procedure could be discussed to avoid major pitfalls and to get a better understanding of the practical challenges in the mouse and how to handle these. This was extremely helpful after own experience was gained with cadaveric procedures. (5), live procedures were performed which were initially very time-consuming but improved quickly with respect to operation times and technical handling. To establish hHTX in mice a well-planned educational program including all these abovenamed steps, and working through them in the mentioned order can help improve the learning process and maximize efficacy and economic premises, as well as reproducibility.

### Technical aspects and trouble shooting

Sharing expertise is an important factor when starting the establishment of the hHTX in mice since the operation itself is technically demanding and small details can be pivotal for increasing success. During training, we were able to learn from other working groups that already established the operation. Therefore, some major pitfalls could be avoided. Nonetheless, we were able to identify several issues that affect the success of the operation and new key factors that further simplify the operation and increase success rates.

- We studied procedural success using mice of different age groups (7–18 weeks) and found no statistically significant difference regarding survival. The technical complexity, however, was lowest for mice aged 8–10 weeks. Older mice had increasing amounts of fatty and fragile tissue while in younger mice the vessel diameter was too small. We did not observe significant differences regarding thrombosis rates regarding mouse age as suggested by Martins.[43]
- We observed a better survival for female mice but more reliable and stable results regarding weight reduction of the unloaded hearts in male mice.
- Technically, the male recipients were more difficult because of the gonadal vessels that frequently need to be dissected off the aorta/IVC.
- The properties of the clips interrupting the blood flow during the anastomoses are essential. After trying different options, we used aneurysm clips (Yasargil Aneurysm Clip System, Aesculap, Inc., Center Valley, PA, USA), since they apply the right amount of pressure, and are very slim. This way retrograde bleeding as well as injury of the vessel wall could be avoided, and the length of vessels for the anastomoses can be maximumised.
- The first and last stitches should be placed close to the stay sutures as the ends of the anastomoses are most vulnerable to bleeding or congestion.
- The venous anastomosis was done with very loose stitches, and a loop suture was applied in the middle of the first side of the anastomosis to prevent congestion.
- To keep the procedure as simple and fast as possible, and to minimize manipulation of the heart, the second side of the venous anastomosis was performed without flipping the heart over again. The suture was pulled through outwardly between the PA and the IVC after the distal anchor stitch was performed. The continuous suture was then started on the right side of the anastomosis, stitching from outside-to-inside and inside-to-outside.
- Intestines should not be removed from the abdominal cavity to avoid torsion, mesenterial ischemia, excessive loss of fluids and loss of body temperature.
- In the beginning, we used cardioplegic solution, to flush the graft at explantation, and as a storage solution during cold ischemia. With these hearts we saw significantly slower onset of spontaneous beating, sometimes the hearts did not start to beat at all, despite technically sound anastomoses. Remnant cardioplegic solution could lead to prolonged and incomplete recovery of the transplanted heart. Accordingly, we used ice-cold saline solution for storage and flushing of the graft.
- When flushing the heart initially during the explantation procedure, the syringe had to be free of air bubbles. Once air bubbles are trapped in the heart it is difficult to prevent air embolism later on.
- Furthermore, flushing of the heart by puncturing the aortic arch before harvesting the donor heart must be done with great care to avoid injury to the aortic valve which results in aortic regurgitation and filling of the left ventricle, thereby preventing mechanical unloading of the graft.
- To increase the overview for the preparation of the ascending aorta and pulmonary artery, the thymus of the donor animal was dissected before harvesting the heart. This could easily be performed by tearing the left and right part apart.
- The pulmonary artery and the IVC were handled very carefully with minimal manipulation to avoiding tearing of the vessel injury which is usually irreparable.
- Intraoperative technical accuracy was in our experience the most relevant factor for the postoperative survival and success. Despite close monitoring and postoperative supportive strategies, intraoperative complications were in most cases irreversible.

In our experience, a training period of two months with constant operations, and a total number of 50–60 operations during this period seems to be appropriate to achieve stable operation times and a good operation routine. Major technical challenges are the ligation of the lumbar vessels in the recipient animal and the creation of the anastomoses. The venous anastomosis in particular requires a sensitive handling and precise placement of sutures.

We believe that the technical success during the transplantation is essential for the postoperative survival and success of the model. Gender analysis revealed a better postoperative survival in female mice, but a tendency to more stable unloading results in male mice. Given the more stable heart weight reduction, male mice could be preferred for unloading studies, despite the poorer survival rates.

### Perioperative management

A structured postoperative management and scoring analysis was introduced during the establishment of the procedure in close cooperation with the in-house animal care facility. The scoring revealed the complexity of this operation and gave important feedback to the surgeon. Primarily, the scoring points relating to general health condition and spontaneous animal behavior corresponded well with the survival during the observation period. Change in body weight did not predict the outcome as this would develop too slowly. Also, breathing or wound healing were not associated with outcome. Interestingly, the outcome of the procedure could not be positively affected by the postoperative supportive strategies. The success of the procedure was determined intraoperatively which supports the importance of operative precision. However, the postoperative scoring had applications to limiting the suffering of animals. As the supportive interventions could not affect the survival of the animals, veterinary care and also euthanasia should be evaluated early when an intermediate stress level (as defined in the scoring) is reached.

## Conclusions

hHTX in mice is an adequate model to study the remodeling of mechanically unloaded hearts. Establishing abdominal hHTX in the mouse, however, is laborious, time-consuming and costly, but can be improved considerably by sharing expertise, a structured programme and avoiding the identified pitfalls presented above.

## Supporting information

S1 Table Learning curve for total operation time (min), warm ischemia time (min), and cold ischemia time (min). Operation time, warm ischemia time, and cold ischemia time for each operation in chronological order are displayed.
(XLSX)

S2 Table Learning curve for success rate. Success was defined by a well-beating donor-heart after transplantation and survival of the recipient ≥48 hours.
(XLSX)

S3 Table Correlation between postoperative total scoring points and survival. A mean value for 10 consecutive operations was calculated.
(XLSX)

S4 Table Correlation between postoperative scoring points for body weight, general health condition, and spontaneous behaviour and survival. Postoperative scoring routine started 24 hours after the operation. Therefore only animals that survived > 24 hours were included in the scoring routine.
(XLSX)

S5 Table Gender distribution over time for each operation. For each consecutive operation the gender is listed.
(XLSX)

## References

[1] Kirklin JK, Pagani FD, Kormos RL, Stevenson LW, Blume ED, Myers SL, et al Eighth annual INTERMACS report: Special focus on framing the impact of adverse events. J Heart Lung Transplant. 2017;36(10):1080–6. 10.1016/j.healun.2017.07.005 28942782

[2] Dipla K, Mattiello JA, Jeevanandam V, Houser SR, Margulies KB. Myocyte recovery after mechanical circulatory support in humans with end-stage heart failure. Circulation. 1998;97(23):2316–22. 9639375 10.1161/01.cir.97.23.2316

[3] Drakos SG, Mehra MR. Clinical myocardial recovery during long-term mechanical support in advanced heart failure: Insights into moving the field forward. J Heart Lung Transplant. 2016;35(4):413–20. 10.1016/j.healun.2016.01.001 26922277

[4] Muthiah K, Humphreys DT, Robson D, Dhital K, Spratt P, Jansz P, et al Longitudinal structural, functional, and cellular myocardial alterations with chronic centrifugal continuous-flow left ventricular assist device support. J Heart Lung Transplant. 2017;36(7):722–31. 10.1016/j.healun.2016.05.017 27373819

[5] Burkhoff D, Holmes JW, Madigan J, Barbone A, Oz MC. Left ventricular assist device-induced reverse ventricular remodeling. Prog Cardiovasc Dis. 2000;43(1):19–26. 10.1053/pcad.2000.7190 10935554

[6] Mancini DM, Beniaminovitz A, Levin H, Catanese K, Flannery M, DiTullio M, et al Low incidence of myocardial recovery after left ventricular assist device implantation in patients with chronic heart failure. Circulation. 1998;98(22):2383–9. 9832482 10.1161/01.cir.98.22.2383

[7] Maybaum S, Mancini D, Xydas S, Starling RC, Aaronson K, Pagani FD, et al Cardiac improvement during mechanical circulatory support: a prospective multicenter study of the LVAD Working Group. Circulation. 2007;115(19):2497–505. 10.1161/CIRCULATIONAHA.106.633180 17485581

[8] Muller J, Wallukat G, Weng YG, Dandel M, Spiegelsberger S, Semrau S, et al Weaning from mechanical cardiac support in patients with idiopathic dilated cardiomyopathy. Circulation. 1997;96(2):542–9. 9244223 10.1161/01.cir.96.2.542

[9] Birks EJ, Tansley PD, Hardy J, George RS, Bowles CT, Burke M, et al Left ventricular assist device and drug therapy for the reversal of heart failure. N Engl J Med. 2006;355(18):1873–84. 10.1056/NEJMoa053063 17079761

[10] Klotz S, Foronjy RF, Dickstein ML, Gu A, Garrelds IM, Danser AH, et al Mechanical unloading during left ventricular assist device support increases left ventricular collagen cross-linking and myocardial stiffness. Circulation. 2005;112(3):364–74. 10.1161/CIRCULATIONAHA.104.515106 15998679

[11] Oriyanhan W, Tsuneyoshi H, Nishina T, Matsuoka S, Ikeda T, Komeda M. Determination of optimal duration of mechanical unloading for failing hearts to achieve bridge to recovery in a rat heterotopic heart transplantation model. J Heart Lung Transplant. 2007;26(1):16–23. 10.1016/j.healun.2006.10.016 17234512

[12] Ito K, Nakayama M, Hasan F, Yan X, Schneider MD, Lorell BH. Contractile reserve and calcium regulation are depressed in myocytes from chronically unloaded hearts. Circulation. 2003;107(8):1176–82. 12615798 10.1161/01.cir.0000051463.72137.96

[13] Soppa GK, Barton PJ, Terracciano CM, Yacoub MH. Left ventricular assist device-induced molecular changes in the failing myocardium. Curr Opin Cardiol. 2008;23(3):206–18. 10.1097/HCO.0b013e3282fc7010 18382208

[14] Soppa GK, Lee J, Stagg MA, Siedlecka U, Youssef S, Yacoub MH, et al Prolonged mechanical unloading reduces myofilament sensitivity to calcium and sarcoplasmic reticulum calcium uptake leading to contractile dysfunction. J Heart Lung Transplant. 2008;27(8):882–9. 10.1016/j.healun.2008.05.005 18656802

[15] Schwoerer AP, Neef S, Broichhausen I, Jacubeit J, Tiburcy M, Wagner M, et al Enhanced Ca(2)+ influx through cardiac L-type Ca(2)+ channels maintains the systolic Ca(2)+ transient in early cardiac atrophy induced by mechanical unloading. Pflugers Arch. 2013;465(12):1763–73. 10.1007/s00424-013-1316-y 23842739 PMC3898408

[16] Schwoerer AP, Neuber C, Schmechel A, Melnychenko I, Mearini G, Boknik P, et al Mechanical unloading of the rat heart involves marked changes in the protein kinase-phosphatase balance. J Mol Cell Cardiol. 2008;45(6):846–52. 10.1016/j.yjmcc.2008.09.003 18848565

[17] Hasegawa T, Visovatti SH, Hyman MC, Hayasaki T, Pinsky DJ. Heterotopic vascularized murine cardiac transplantation to study graft arteriopathy. Nat Protoc. 2007;2(3):471–80. 10.1038/nprot.2007.48 17406609

[18] Niimi M. The technique for heterotopic cardiac transplantation in mice: experience of 3000 operations by one surgeon. J Heart Lung Transplant. 2001;20(10):1123–8. 11595568 10.1016/s1053-2498(01)00309-6

[19] Gong W, Liu B, Chen J, Liu C, Shen Z. Impact of Regulatory T Cells on Innate Immune Cells in a Pre-Sensitized Heart Transplant Model. Ann Transplant. 2018;23:246–51. 10.12659/AOT.907598 29650945 PMC6248278

[20] Habertheuer A, Korutla L, Rostami S, Reddy S, Lal P, Naji A, et al Donor tissue-specific exosome profiling enables noninvasive monitoring of acute rejection in mouse allogeneic heart transplantation. J Thorac Cardiovasc Surg. 2018;155(6):2479–89. 10.1016/j.jtcvs.2017.12.125 29499866

[21] Sharma M, Liu W, Perincheri S, Gunasekaran M, Mohanakumar T. Exosomes expressing the self-antigens myosin and vimentin play an important role in syngeneic cardiac transplant rejection induced by antibodies to cardiac myosin. Am J Transplant. 2018;18(7):1626–35. 10.1111/ajt.14650 29316217 PMC6035065

[22] Hartung D, Hueper K, Chen R, Gutberlet M, Wacker F, Meier M, et al T2 Mapping for Noninvasive Assessment of Interstitial Edema in Acute Cardiac Allograft Rejection in a Mouse Model of Heterotopic Heart Transplantation. Invest Radiol. 2018;53(5):271–7. 10.1097/RLI.0000000000000438 29261532

[23] Yang B, He F, Dai C, Tan R, Ma D, Wang Z, et al BATF inhibition prevent acute allograft rejection after cardiac transplantation. Am J Transl Res. 2016;8(8):3603–13. 27648151 PMC5009413

[24] Lu C, Zeng YQ, Liu H, Xie Q, Xu S, Tu K, et al Tanshinol suppresses cardiac allograft rejection in a murine model. J Heart Lung Transplant. 2017;36(2):227–36. 10.1016/j.healun.2016.07.016 27574736

[25] Zhang A, Wang K, Zhou C, Gan Z, Ma D, Ye P, et al Knockout of microRNA-155 ameliorates the Th1/Th17 immune response and tissue injury in chronic rejection. J Heart Lung Transplant. 2017;36(2):175–84. 10.1016/j.healun.2016.04.018 27296836

[26] Chatur S, Wong BW, Carthy JM, McManus BM. Inhibition of vascular endothelial growth factor reduces cardiac allograft vasculopathy. J Heart Lung Transplant. 2016;35(9):1124–30. 10.1016/j.healun.2016.04.011 27266812

[27] Cai S, Ichimaru N, Zhao M, Fujino M, Ito H, Ota U, et al Prolonged Mouse Cardiac Graft Cold Storage via Attenuating Ischemia-Reperfusion Injury Using a New Antioxidant-Based Preservation Solution. Transplantation. 2016;100(5):1032–40. 10.1097/TP.0000000000001079 26845308

[28] Dare AJ, Logan A, Prime TA, Rogatti S, Goddard M, Bolton EM, et al The mitochondria-targeted anti-oxidant MitoQ decreases ischemia-reperfusion injury in a murine syngeneic heart transplant model. J Heart Lung Transplant. 2015;34(11):1471–80. 10.1016/j.healun.2015.05.007 26140808 PMC4626443

[29] Brunner SM, Schiechl G, Falk W, Schlitt HJ, Geissler EK, Fichtner-Feigl S. Interleukin-33 prolongs allograft survival during chronic cardiac rejection. Transpl Int. 2011;24(10):1027–39. 10.1111/j.1432-2277.2011.01306.x 21797940

[30] Schiechl G, Brunner SM, Kesselring R, Martin M, Ruemmele P, Mack M, et al Inhibition of innate co-receptor TREM-1 signaling reduces CD4(+) T cell activation and prolongs cardiac allograft survival. Am J Transplant. 2013;13(5):1168–80. 10.1111/ajt.12186 23463907

[31] Abbott CP, Lindsey ES, Creech O Jr., Dewitt CW. A Technique for Heart Transplantation in the Rat. Arch Surg. 1964;89:645–52. 14186795 10.1001/archsurg.1964.01320040061009

[32] Ono K, Lindsey ES. Improved technique of heart transplantation in rats. J Thorac Cardiovasc Surg. 1969;57(2):225–9. 4884735

[33] Plenter RJ, Grazia TJ. Murine heterotopic heart transplant technique. J Vis Exp. 2014(89). 10.3791/51511 PMC4212889 25046118

[34] Plenter RJ, Zamora MR, Grazia TJ. Four decades of vascularized heterotopic cardiac transplantation in the mouse. J Invest Surg. 2013;26(4):223–8. 10.3109/08941939.2012.755238 23514056

[35] Fang J, He L, Wang SQ, Ma MJ, Liu HY, Zhu XH, et al A simplified two-stitch sleeve technique for arterial anastomosis of cervical heterotopic cardiac transplantation in mice. Am J Transl Res. 2013;5(5):521–9. 23977411 PMC3745439

[36] Ratschiller T, Deutsch MA, Calzada-Wack J, Neff F, Roesch C, Guenzinger R, et al Heterotopic Cervical Heart Transplantation in Mice. J Vis Exp. 2015(102):e52907 10.3791/52907 26325193 PMC4692555

[37] Wang C, Wang Z, Allen R, Bishop GA, Sharland AF. A modified method for heterotopic mouse heart transplantion. J Vis Exp. 2014(88):e51423 10.3791/51423 24998365 PMC4204913

[38] Li C, Luo L, Lu J, Feng L, Shan J, Long D, et al A modified splint tubing technique for heterotopic heart transplantation in mouse. Transpl Immunol. 2011;25(1):82–7. 10.1016/j.trim.2011.03.005 21513800

[39] Mao M, Liu X, Tian J, Yan S, Lu X, Gueler F, et al A novel and knotless technique for heterotopic cardiac transplantation in mice. J Heart Lung Transplant. 2009;28(10):1102–6. 10.1016/j.healun.2009.05.025 19782294

[40] Corry RJ, Winn HJ, Russell PS. Primarily vascularized allografts of hearts in mice. The role of H-2D, H-2K, and non-H-2 antigens in rejection. Transplantation. 1973;16(4):343–50. 4583148 10.1097/00007890-197310000-00010

[41] Didie M, Biermann D, Buchert R, Hess A, Wittkopper K, Christalla P, et al Preservation of left ventricular function and morphology in volume-loaded versus volume-unloaded heterotopic heart transplants. Am J Physiol Heart Circ Physiol. 2013;305(4):H533–41. 10.1152/ajpheart.00218.2013 23771692

[42] Schwoerer AP, Melnychenko I, Goltz D, Hedinger N, Broichhausen I, El-Armouche A, et al Unloaded rat hearts in vivo express a hypertrophic phenotype of cardiac repolarization. J Mol Cell Cardiol. 2008;45(5):633–41. 10.1016/j.yjmcc.2008.02.271 18721926

[43] Martins PN. Assessment of graft function in rodent models of heart transplantation. Microsurgery. 2008;28(7):565–70. 10.1002/micr.20544 18767132

